# The potential interaction between clopidogrel and proton pump inhibitors: a systematic review

**DOI:** 10.1186/1741-7015-8-81

**Published:** 2010-12-06

**Authors:** Joao Paulo de Aquino Lima, James M Brophy

**Affiliations:** 1 Federal University of Ceará School of Medicine, Fortaleza, Brazil; 2 Department of Medicine, McGill University, Montreal, Quebec, Canada

## Abstract

**Background:**

Recently, several publications have investigated a possible drug interaction between clopidogrel and proton pump inhibitors (PPIs), and regulatory agencies have issued warnings despite discordant study results. In an attempt to clarify the situation, we performed a systematic review with a critical analysis of study methodologies to determine whether varying study quality (that is, bias) could explain the discordant results.

**Methods:**

A systematic review of all studies reporting clinical outcomes was performed using an electronic literature search of the MEDLINE and EMBASE databases, abstracts from the major cardiology conferences and a hand-search of bibliographies from identified articles. Each study was evaluated for its risk of bias according to a prespecified quality measure scale.

**Results:**

A total of 18 studies were identified. Ten of 13 studies judged to be of low scientific quality reported a statistically positive interaction between clopidogrel and the general class of PPIs, and each concluded this was likely a clinically meaningful effect. None of the five studies judged to be of moderate or high quality reported a statistically significant association. Multiple sources of heterogeneity (that is, different populations, outcomes assessed, drug exposure methods and study quality) prevented a formal quantitative analysis of all studies. An increased risk of bias was observed in the positive studies, resulting in an inverse correlation between study quality and a reported statistically positive interaction (10/13 versus 0/5; *P *= p = 0.007). There was also no clinical evidence for a positive interaction according to specific PPIs.

**Conclusion:**

The observed association between clopidogrel and PPIs is found uniquely in studies judged to be of low quality and with an increased risk of bias. High-quality evidence supporting a clinically significant clopidogrel/PPI interaction is presently lacking.

## Background

Clopidogrel is a widely prescribed thienopyridine for the prevention of atherothrombotic complications following acute coronary syndromes (ACS) or percutaneous coronary interventions (PCIs) [1]. Clopidogrel is a prodrug that has no intrinsic antiplatelet activity without activation by hepatic metabolism through the cytochrome P450 (CYP) system [2]. Multiple CYP enzymes have been implicated in this process, but recently the CYP2C19 enzyme has assumed predominance as it is involved in both sequential oxidative steps [3]. The possibility of drug interactions limiting clopidogrel's efficacy was raised several years following *in vitro *statin and clopidogrel studies, but without definitive clinical confirmation of increased adverse outcomes [4-6]. Recently, mechanistic *in vitro *studies have suggested that proton pump inhibitors (PPIs) may diminish clopidogrel's clinical efficacy via CYP2C19 competitive inhibition [7-10]. Consistent with these *in vitro *observations, several clinical studies have shown higher cardiovascular events in clopidogrel patients exposed to PPIs compared to those not exposed [11,12], leading the Food and Drug Administration (FDA) [13] and the European Medicines Agency [14] to issue public alerts recommending the avoidance of prescribing PPIs in patients who also take clopidogrel. However, as the studies have largely been nonexperimental, the possibility of a spurious association due to bias needs to be attentively considered.

This is a clinically important question as many cardiac patients are also at high risk of gastrointestinal (GI) bleeding (due to age, smoking and concomitantly prescribed drugs), and PPIs may substantially mitigate this risk [15]. Previous reviews of this interaction question have appeared [16-20], but they have not (1) been systematic, (2) provided a critical analysis of methodological issues or (3) integrated these safety concerns with prior knowledge of clopidogrel's time frame of action. The present review addresses these issues by performing a systematic and critical analysis of all clinical studies of this putative interaction.

## Methods

We reviewed the MEDLINE and EMBASE electronic databases from January 1, 2005, to October 7, 2010, without any language restriction, combining search terms for clopidogrel ("clopidogrel" OR "Plavix" OR "thienopyridine"), PPIs ("PPI" OR " omeprazole" OR "lansoprazole" OR "pantoprazole" OR "esomeprazole" OR "rabeprazole") with those for cardiovascular outcomes ("mortality" OR "cardiovascular disease" OR "heart disease" OR "CAD" OR "MI" OR "UA" OR "coronary angiography" OR "coronary restenosis" OR "PCI" OR "stroke") and drug interaction ("interaction" OR " inhibition" OR " CYP2C19"). References of relevant identified articles were hand-searched for additional studies. Abstracts from medical organization conferences (American Heart Association, American College of Cardiology, European Society of Cardiology and Transcatheter Cardiovascular Therapeutics) were manually searched from 2005. *A priori *it was decided that only articles reporting clinical outcomes would be retained. Mechanistic *in vitro *studies measuring platelet aggregation were therefore excluded. Two investigators independently reviewed articles for inclusion and study quality. Both reviewers examined the methodology component independently of the study results, but given the publicity surrounding the individual studies it was impossible to ensure that the reviewers were totally blinded to the outcomes. It was planned to resolve disagreements by consensus, but there was perfect agreement between the investigators on their initial assessments. This systematic review was performed according to the PRISMA guidelines (see online Additional file 1).

Overall study quality was defined as high for *well-performed *randomized clinical trials (RCTs) as they exhibit the best internal validity. Observational studies are considered of lower quality as they have less internal validity due to numerous potential biases and consequently could attain a maximum moderate quality score. By following published guidelines to improve the reporting of observational studies [21] and specifically to mitigate against potential biases [22], we qualitatively evaluated all the observational studies for their propensity for the most common and important biases (selection, confounding and misclassification), thereby allowing their final classification into moderate- or low-quality levels. Overall observational study quality was judged to be moderate if the propensity for bias was low and specific methods to minimize bias were employed (explicit consideration of the time dependency of drug exposure and appropriate clinical outcome assessment to minimize misclassification, multiple methods to assess confounding and multivariate sensitivity analyses to test the independence and robustness of their results). Alternatively, studies were judged to be at moderate risk for bias and hence a low quality score.

The quality score of unpublished studies was systematically reduced by one grade owing to the absence of formal peer review. Isolated abstracts were considered to be of low quality as there are insufficient details to fully evaluate these reports. Finally, whenever possible, an external quality measure that compared survival curves from the clopidogrel efficacy randomized trials to the observational hazard ratios was performed to check for consistency in the etiologically relevant time windows.

Since study heterogeneity existed with respect to research methods, quality, study populations and health care systems, it was decided *a priori *that quantitative data pooling was inappropriate.

## Results

**Figure 1:**
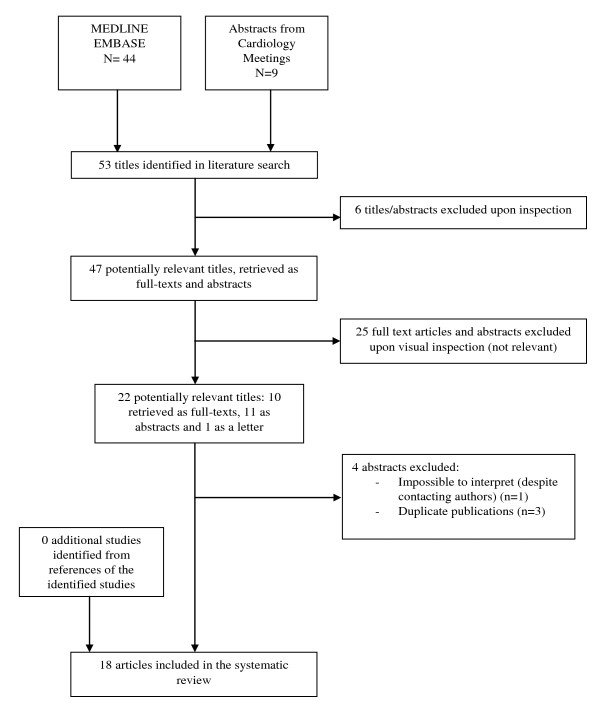
**Flow diagram of selection process of studies included in the systematic review**.

Our literature search found 54 publications, and 18 of these studies [11,12,23-38] fulfilled our inclusion criteria (see Figure 1). One study was experimental with random PPI allocation [25], while the others were nonexperimental. Study details are presented in Table 1. Two studies [12,36] with an otherwise low propensity for bias considered a primary endpoint of only nonfatal myocardial infarction (MI), ignoring fatal myocardial infarction, which has been a recurrent component of the composite measure of clopidogrel efficacy. Although an increased risk with the clopidogrel-PPI combination for nonfatal myocardial infarction was observed in both studies, mortality was actually lower in the PPI-exposed group in one study [12] and was not reported in the other [36], such that the more logical and standard combined endpoint of cardiovascular death and nonfatal MI could not be reliably evaluated. The lack of justification for their primary outcome and lack of sensitivity analyses regarding potential different adverse outcomes explains the lower assigned quality ratings for these two studies.

**Table 1:** Study detailsa

Study	Population (special conditions)	PPIs studied	Propensity for bias*	Procedures to minimize bias**	Study quality***	Primary outcome/Results (all PPIs combined)	Secondary analyses according to specific PPIs	Authors' conclusions about clopidogrel-PPI interaction
Ho *et al*. [11]	8,205 ACS (VA patients)	L, O, P, R	Moderate	Yes	Low	3-year mortality or ACS OR: 1.25; 95% CI: 1.11-1.41	O = OR: 1.24; 95% CI: 1.08-1.41 R = OR, 2.83; 95% CI: 1.96-4.09 L NR P NR	"Concomitant use after hospital discharge for ACS was associated with increased risk of adverse outcomes."
Juurlink *et al*. [12]	13,636 ACS (age ≥66)	L, O, P, R	Moderate	Yes	Low	90-day nonfatal MI OR: 1.27; 95% CI: 1.03-1.57	P = OR 1.02, 95% CI 0.70-1.47 Non P = OR 1.40 (1.10-1.77)	"Concomitant therapy with PPIs other than pantoprazole was associated with loss of beneficial effects of clopidogrel."
Aubert *et al*. [23]	14,383 PCI	NR	Uncertain	Uncertain	Low	1-year combined stroke, MI, angina or CABG OR:1.79; 95% CI 1.62-1.97	NR	"The drug interaction between PPIs and clopidogrel may result in serious adverse outcomes within one year of therapy initiation."
Banerjee *et al*. [24]	534 PCI	NR	Uncertain	Uncertain	Low	Mortality, MI, repeat revascularization and stroke OR 1.54 no CI *P *< 0.006 (557-615 days)	NR	"PPI(s) with clopidogrel is associated with an increased risk."
Bhatt *et al*. [25]	3,267 ACS PCI	O	Low	Yes	High	130-day CD, nonfatal MI, revascularization or ischemic stroke HR: 1.02; 95% CI = 0.70-1.51	Only O studied	"No apparent cardiovascular interaction between clopidogrel and omeprazole"
Dunn *et al*. [26]	1,053 PCI	NR	Uncertain	No	Low	1-year death, MI or stroke OR: 1.63; 95% CI, 1.02-2.62	NR	"PPI use was associated with an increased cardiovascular risk."
Gaspar *et al*. [27]	922 ACS	O, L, R	Uncertain	Uncertain	Low	6-month death, MI or UA 8.8% vs. 8.4%, *P *= NS	NR	"Was not associated with a worse prognosis in patients with ACS"
Gupta *et al*. [28]	315 PCI (VA patients)	L, O, R	Moderate	No	Low	4-year death, MI or TVF OR: 1.95; 95% CI: 1.09-3.49	NR	"PPIs may attenuate clopidogrel's beneficial antiplatelet effect."
Huang *et al*. [29]	3,278 PCI (Taiwan)	NR	Moderate	No	Low	5-year all-cause mortality HR: 1.65; 95% CI, 1.35-2.01	NR	"Concomitant use should be done with care to avoid adverse outcomes."
O'Donoghue *et al*. [30]	6,795 ACS (age ≥60)	E, L, O, P, R	Low	Yes	Moderate	1-year CD, MI or stroke OR: 0.94; 95% CI: 0.80-1.11	No specific data reported "Regardless of PPI type (including omeprazole alone or the exclusion of pantoprazole), no independent association existed"	"No clinically relevant adverse cardiovascular interaction between clopidogrel and PPIs."
Pezalla *et al*. [31]	1,010 ACS (age <65)	NR	Uncertain	No	Low	1-year adjusted MI OR 4.3; 95% CI 2.2-8.4^#^	NR	"Evidence is pointing toward a potentially significant interaction."
Ramirez *et al*. [32]	534 PCI	NR	Uncertain	Uncertain	Low	1-year mortality/MI OR NA *P *= 0.32	NR	"Concomitant use of PPI's did not result in adverse cardiovascular outcomes at one year."
Rassen *et al*. [33]	18,565 ACS PCI (age ≥65)	E, L, O, P, R	Low	Yes	Moderate	180-day death or MI OR: 1.22; 95% CI: 0.99-1.51	O = RR 1.17; 95% CI: 0.68-2.01 P = RR 1.26; 95% CI: 0.93-1.71	"We did not observe conclusive evidence of a clopidogrel/PPI interaction."
Ray *et al*. [34]	20,596 ACS PCI (age ≥30)	E, L, O, P, R	Low	Yes	Moderate	1-year MI, CD or stroke HR: 0.99; 95% CI, 0.82-1.19	E = HR 0.71; 95% CI 0.48-1.06 L = HR 1.06; 95% CI 0.77-1.45 O = HR 0.79;95% CI 0.54-1.15 P = HR 1.08; 95% CI 0.88-1.32 R = HR 0.54; 95% CI 0.30-0.97	"Concurrent use of a PPI was not associated with a statistically significant increased risk for serious cardiovascular disease."
Sarafoff *et al*. [35]	2,025 PCI	NR	Uncertain	Uncertain	Low	30-day stent thrombosis OR NR, *P *= 0.002 death OR NR, *P *= 0.04	NR	"A PPI is associated with higher rates of stent thrombosis and an increased mortality."
Stockl *et al*. [36]	2,066 ACS PCI (age 18-84)	E, L, O, P, R	Moderate	Yes	Low	1-year nonfatal MI HR: 1.93; 95% CI: 1.05-3.54	P = HR 2.18; 95% CI 0.88-5.39	"Patients who received clopidogrel plus a PPI had a significantly higher risk."
Tsiaousis *et al*. [37]	612 PCI	NR	Uncertain	Uncertain	Low	1-year death HR: 1.1; 95% CI: 0.7-1.4 1-year MI HR: 1.0; 95% CI: 0.8-1.3	NR	"PPIs drug therapy does not have any impact on the effectiveness."
Charlot *et al*. [38]	56,408 AMI	E, L, O, P	Low	Yes	Moderate	1-year death, MI, stroke HR: 0.98; 95% CI, 0.88-1.10	"No difference in risk associated with the type of PPI"	"No statistically significant interaction occurred between a PPI and clopidogrel."

^a^ACS, acute coronary syndrome; AMI, acute myocardial infarction; AR, absolute risk; C, clopidogrel; CABG, coronary artery bypass graft; CD, cardiovascular death; CI, confidence interval; HR, hazard ratio; MI, myocardial infarction; OHIP, Ontario health insurance program; OR, odds ratio; PCI, percutaneous coronary intervention; PPI, proton pump inhibitor; TIA, transient ischemic attack; TVF, target vessel failure; TVR, target vessel revascularization; VA, Veterans Administration; UA, unstable angina; NR, not reported; E, esomeprazole; L, lansoprazole; O, omeprazole; P, pantaprazole; R, rabeprazole.

*Biases considered are selection, confounding and misclassification; the probability of bias in the studies available as abstracts was deemed uncertain.

**Procedures to minimize bias include multiple sensitivity analyses for varying drug exposures, multiple methods for control of confounding and time-varying analysis for misclassification.

***High study quality was reserved for RCTs. For observational studies, moderate study quality required low propensity for bias and specific efforts to minimize bias. Otherwise, quality was assessed as low.

^#^Calculated by combining high- and low-dose PPI groups.

Among the full published studies judged to be methodologically weak, several potential biases (selection, residual confounding, immortal time and interpretative) were noted. Ten of 13 studies judged to be of low quality reported a statistically significant association for a harmful clopidogrel-PPI interaction, while none of the five moderate-quality studies found an association (*P *= 0.007).

Each individual study evaluated a combined exposure to any PPI for the assessment of their primary endpoint, with the exception of the experimental study [25], which was restricted to omeprazole. One study [12] did report a difference between pantoprazole and nonpantoprazole PPIs in their association with adverse clinical outcomes, but the corrected proper statistical analysis actually revealed no difference [39]. No other study demonstrated a clinically significant difference in clinical outcomes among the different PPIs.

## Discussion

Our systematic review identified 18 clinical studies of a clopidogrel-PPI interaction, with the majority (10) reporting a statistically significant result. Multiple sources of heterogeneity (different populations, outcomes assessed, drug exposure methods and study quality) existed between the studies, preventing a formal quantitative analysis of all studies. However, a stratified analysis based on study quality demonstrated an inverse correlation between study quality and a positive outcome. Moreover, none of the 18 studies found a clinically meaningful difference between the various PPIs. Therefore, high-quality evidence supporting a clinically significant clopidogrel-PPI interaction is presently lacking. Consequently, while good prescribing patterns are to be endorsed for all medications, recent edicts to avoid PPIs in cardiac patients with a clinical indication for their use seem poorly justified.

**Figure 2:**
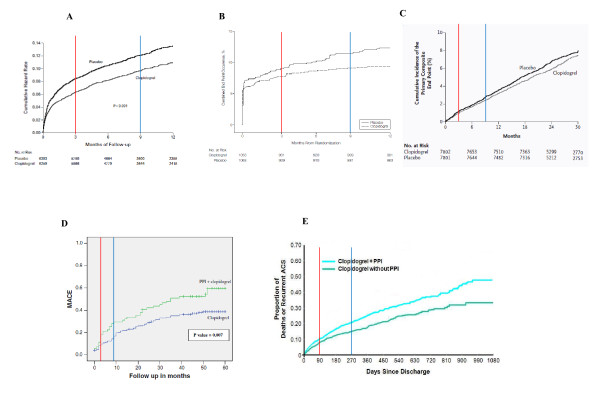
**(A) Survival curves for the primary outcome (cardiovascular death, nonfatal myocardial infarction, or stroke) in randomized trial **[1]**comparing clopidogrel to placebo following an acute coronary syndrome**. **(B) **Survival curves for the primary outcome (cardiovascular death, nonfatal myocardial infarction, or stroke) in randomized trial [39] comparing clopidogrel to placebo following PCI. **(C) **Survival curves for the primary end point (cardiovascular death, myocardial infarction, or stroke) in randomized trial [40] comparing clopidogrel to placebo in patients with clinically evident stable cardiovascular disease or multiple risk factors stable cardiac patients. **(D) **Survival curves for primary outcome (mortality, non-fatal MI and target vessel revascularization) from an observational study [28] comparing PCI patients discharged on clopidogrel plus proton pump inhibitor (PPI) and clopidogrel alone. **(E) **Survival curves for the primary end point (death acute coronary syndrome) from an observational study [11] comparing ACS patients discharged on clopidogrel plus proton pump inhibitor (PPI) and clopidogrel alone. The randomized trials following an ACS or PCI (Figures 2A and 2B) show that the benefit of clopidogrel is obtained early (see red arrow at 3 months) with no or little additional benefit accrued thereafter (see blue arrow at 9 months). The lack of long term benefit in stable patients was confirmed in another randomized trial (Figure 2C). This is contrasted with the results of observational studies (Figures 2D and 2E) where there appears to be no increased risk with PPIs in the short term and the differences arise in the long term where the benefits of clopidogrel have not been demonstrated.

While our criteria for judging study quality are standardized and were applied *a priori *and independently of the study results, their validity would be enhanced by some empirical assessment. Certainly, these quality criteria have face and content validity. In judging the presence of construct validity, it may be helpful to review what is known about clopidogrel efficacy from high-quality clinical trials. The Clopidogrel in Unstable Angina to Prevent Recurrent Events Trial (or CURE) was the seminal randomized trial [1] showing the benefits of clopidogrel in acute coronary syndromes, namely, a 1.9% absolute reduction in the primary endpoint of cardiovascular death, nonfatal MI or stroke with clopidogrel which was evident by 3 months with no or very little additional advantage at later time points (see Figure 2). In a PCI trial [40], clopidogrel again was shown to exhibit its beneficial results principally in the first 30-90 days. A lack of long-term benefit in the non-acute setting was confirmed in the randomized CHARISMA trial [41], which showed no advantages in stable cardiac or high-risk patients at any time over a 28-month follow-up period. Contrast these results with those from the observational studies of the putative drug interaction attributing 9% [11] and 18% [28] absolute increases in adverse outcomes to the inhibition of the protective effects of clopidogrel. Another study [29] attributed a greater than 15% absolute difference in all-cause mortality to this putative association. This represents a many-fold larger effect than the observed clopidogrel benefits in randomized trials. Moreover, in these studies, no significant difference was seen between the clopidogrel-PPI group and the clopidogrel-alone group within the therapeutically active 30-to 90-day window (see Figure 2). The observed adverse effects attributed to the drug interaction were therefore occurring at a time when clopidogrel has not been shown to be therapeutically active. It therefore seems likely that the observed differences between PPI users and nonusers are unrelated to clopidogrel inhibition, but rather are related to residual confounding from unmeasured prognostic variables as measured baseline prognostic factors were markedly different between the groups. Supporting this hypothesis, a *post hoc *analysis of the CREDO study [26] showed a 2% absolute increase in adverse cardiac events even among placebo patients who were exposed to PPIs. Unfortunately, the other observational studies have not presented survival functions, so a similar evaluation of the biological plausibility and consistency of their results cannot be made.

It bears repeating that the only high-quality randomized trial permitting an unconfounded assessment of a causal association [25] did not show any evidence of harm. Moreover, it may be argued that *post hoc *analyses of clinical trial data offer better internal validity than other observational studies because of a greater homogeneity in the patient population, increased and higher-quality baseline clinical data and better outcome validation, thereby potentially mitigating, but certainly not eliminating, possible biases [22]. Our evaluation of the present studies empirically confirmed this association, with the *post hoc *clinical trial [30] receiving a higher score by our independent criteria providing further external support of our quality scale. Again, this higher-quality study did not show evidence for the putative interaction.

Our systematic review provides a reproducible, prespecified and comprehensive review of the totality of the evidence in the literature of a potential interaction between clopidogrel and PPIs, thereby facilitating informed decision making. However, our systematic review may be criticized for our inability to ensure total blinding of methodological assessment from outcomes. As with any systematic review, we are also limited by the quality of the original publications. While we found a paucity of high-quality clinical evidence to support a clopidogrel-PPI interaction either collectively or individually, the power to find such an association is limited and does not preclude the possibility that later high-quality studies may appear to confirm this putative drug association.

The issue of adverse outcomes associated with concomitant use of clopidogrel and PPIs has been assessed in previous reviews [16-20], as well as by American and European regulatory agencies [13,14], which concluded that the interaction is clinically significant. However, previous reviews have been dominated by studies with surrogate laboratory endpoints, were not systematic in identifying the clinical evidence, did not consider the putative interaction effect as a function of our previous knowledge of clopidogrel efficacy and did not provide a critical analysis of the methodological issues of each study. Lower-quality observational studies tend to overestimate effect size because of bias [42]. In the case of regulatory agencies, the threshold to report putative clinical adverse effects is naturally low in an effort to protect public health. However, with the recent spate of controversies regarding the cardiovascular safety of approved drugs such as rofecoxib and rosiglitazone, there is perhaps a strong desire for these agencies to avoid appearing inactive and rather to be perceived as proactive. Nevertheless, it is disconcerting that the potential positive benefits of avoiding gastrointestinal bleeds in high-risk patients appear to have been minimized if not ignored and that regulatory decisions have been made not on a systematic assessment of the clinical evidence, but rather largely on surrogate laboratory data and isolated clinical studies of questionable validity. One regulatory agency [14] has targeted specific PPIs as being culpable without a single valid clinical study to support this claim. Correctly or not, the impression is that the safety of the regulatory agencies from criticism has become more important than the critical assessment of public health safety.

Beyond our specific conclusion that the evidence for a clinically meaningful clopidogrel-PPI interaction is poor, our critical analysis of this potential drug interaction leads to several general observations regarding pharmacoepidemiological research and subsequent policy decisions. First, selection bias or confounding by indication remains a potential threat to all observational research of both intended and unintended effects. Second, residual confounding is likely to be persistent when multivariate analysis is limited to data routinely collected from purely administrative data sets lacking the necessary clinical granularity. *Post hoc *analyses of randomized trials are similarly subject to the same potential biases, but the magnitude may be less owing to both a more homogeneous source population (from the strict inclusion and exclusion criteria, which favor the recruitment of populations with only one major health issue), the availability of more extensive baseline clinical data (decreasing residual confounding) and more systematic outcome assessment through adjudication (decreasing misclassification or information bias), thereby allowing a more rigorous assessment of the independent contribution of any putative association. Third, studies of surrogate endpoints (for example, platelet inhibition) may provide interesting avenues for future outcomes research, but these studies should not be used for clinical and regulatory decision making. The perils of surrogate markers have been repeatedly demonstrated in cardiovascular medicine, with specious examples ranging from ventricular ectopy suppression to lowering of glycosylated hemoglobin. Fourth, when possible, cohort studies should present survival functions, which may help in determining the reasonableness of conclusions by ensuring that events occur within the etiologically relevant time windows as demonstrated by RCTs. Finally, the time-varying nature of drug exposure must be meticulously tracked and sensitivity analyses must be performed to assess the robustness of conclusions to residual confounding or measurement error.

Readers should reasonably expect that the peer review process provides some guidance and discussion about potential biases in observational research. Too often there is a tendency to explain discordant study results not by considering biases, but by assuming that the results are due to different populations, interventions or outcomes [43,44]. In our opinion, the probability of such effect modification is often small compared to the probability of bias. While some biases are subtle, others become more obvious with attentive reading and consideration of the totality of the evidence. The need to improve the reliability and value of medical research has been well recognized by many groups [43-45], and several guidelines have been published [21,22], including those that pertain to observational research, but more specific guidelines for designing and interpreting pharmacoepidemiological studies may be helpful.

## Conclusions

This systematic review of the putative drug interaction between clopidogrel and PPIs identified 18 clinical studies. Rigorous and extensive evaluation of the study methodologies demonstrated that the majority were of low quality, and only these studies reported a positive association between clopidogrel-PPI exposure and increased cardiovascular events. Supporting the notion that bias may be responsible for a spurious association, the increase in cardiovascular events attributed to this interaction appears to be occurring within a therapeutic window when clopidogrel has not been shown to be etiologically active. Conversely, all studies assessed as high quality did not demonstrate this association. No study was able to isolate any individual PPI as being more prone to adverse cardiac outcomes. Careful attention to the methodological details of published studies is therefore mandatory to avoid reaching questionable scientific conclusions. In light of this systematic review, and given the potential benefit of PPIs for patients at high risk of gastrointestinal bleeding, regulatory agencies may wish to reevaluate their recent edicts regarding this putative interaction.

## Competing interests

The authors declare that they have no competing interests.

## Authors' contributions

JMB conceived the project. JMB and JPAL developed the protocol jointly and independently abstracted the data. JMB did the statistical analysis. JMB and JPAL jointly wrote all drafts and the final manuscript.

## Pre-publication history

The pre-publication history for this paper can be accessed here:

http://www.biomedcentral.com/1741-7015/8/81/prepub

## Supplementary Material

Additional file 1 **PRISMA Checklist**. This file is recommended to be included in all systematic review. The checklist enables the reader to rapidly identify where the key elements required in a high-quality systematic review are found. Click here for file
